# Limonin ameliorates indomethacin‐induced intestinal damage and ulcers through Nrf2/ARE pathway

**DOI:** 10.1002/iid3.787

**Published:** 2023-02-25

**Authors:** Bo Jia, Leyi Zhao, Pengpeng Liu, Meng Li, Zhilei Tian

**Affiliations:** ^1^ Department of Spleen and Stomach Diseases, Dongzhimen Hospital Beijing University of Chinese Medicine Beijing China; ^2^ Qihuang College Beijing University of Chinese Medicine Beijing China; ^3^ School of Pharmacy Liaoning University of Traditional Chinese Medicine Dalian China; ^4^ Department of Gastroenterology Air Force Specialty Medical Center Beijing China

**Keywords:** INDO, inflammation, intestinal damage and ulcers, Limonin, Nrf2/ARE pathway, oxidative stress

## Abstract

**Background:**

Nonsteroidal anti‐inflammatory drugs (NSAIDs) can cause intestinal damage and ulcers and the incidence is increasing. Limonin plays an important role in the regulation of inflammatory diseases, but it has not been reported in the treatment of intestinal injury and ulcers.

**Methods:**

Indomethacin (INDO) induced intestinal injury and ulcer model in rats. The indexes related to intestinal injury were detected. Western blot and molecular docking techniques were used to detect the docking between Limonin and Nrf2. Next, ML385, an inhibitor of Nrf2/ARE signaling pathway, was applied to treat intestinal epithelial IEC‐6 cells induced by INDO. And CCK8, Western blot, TUNEL, ELISA, DCFH‐DA assay, kits, and immunofluorescence were conducted to detect cell activity, apoptosis, inflammatory response, oxidative stress, and tight junction again.

**Results:**

INDO can significantly induce intestinal ulcerative lesions in rats. Limonin could improve intestinal ulcerative lesions induced by INDO in rats. Limonin could reduce INDO‐induced inflammatory response and oxidative stress in the small intestine of rats, and improve the intestinal barrier dysfunction induced by INDO. Limonin could dock with Nrf2 structure and activate Nrf2/ARE signaling pathway. ML385 could reverse the protective effect of Limonin against INDO‐induced cell damage.

**Conclusion:**

Limonin ameliorates INDO‐induced intestinal damage and ulcers through Nrf2/ARE pathway.

## Graphical abstract

Nonsteroidal anti‐inflammatory drugs (NSAIDs) can cause intestinal damage and ulcers and the incidence is increasing. Limonin plays an important role in the regulation of inflammatory diseases. In this study, we concluded that Limonin ameliorates INDO‐induced intestinal damage and ulcers through Nrf2/ARE pathway.

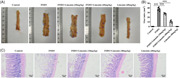

## 1 INTRODUCTION

Nonsteroidal anti‐inflammatory drugs (NSAIDs), including indomethacin (INDO), are widely used in clinic because of their anti‐inflammatory, analgesic, and antipyretic effects.^1^ However, clinical observation has shown that NSAIDs exhibit a variety of clinical side effects while exerting their pharmacological effects, the most common of which is gastrointestinal ulcers and damage.^2^ With the increasing dosage of INDO, the incidence of NSAIDS‐related intestinal injury and ulcer is bound to rise. The prevention of intestinal injury has become an urgent clinical problem.

Active components of natural products have become an important source of drug development due to their diverse skeletal structures and extensive biological activities.^3^ These innovative drugs discovered from natural products have been widely used in clinical practice due to their definite efficacy and few side effects.^4^ There are many studies on the effects of synthetic and natural compounds on gastrointestinal ulcers.^5^, ^6^ Therefore, structural modification of active natural products to find and develop innovative drugs is a feasible way. Limonin, one of the active ingredients of floris sophorae powder, has been safely used as a nutritional and health‐promoting dietary supplement. Limonin improves ulcerative colitis by regulating STAT3/miR‐214 signaling pathway in IL‐6‐induced normal colonic epithelial cells NCM460 cells.^7^ Limonin improves chronic colitis induced by glucan sulfate in mice by inhibiting endoplasmic reticulum stress in colon tissues.^8^ In addition, oral Limonin can up‐regulate the amino acid metabolism, lipid metabolism, and immune system function. And Limonin also inhibits predictive markers of immune system diseases and infectious diseases. In the infectious disease category, markers of bacterial toxins and *Staphylococcus aureus* infection were significantly inhibited after Limonin treatment.^9^ However, the effect of Limonin on INDO‐induced intestinal ulcer and injury has not been reported so far.

Previous study has shown that Limonin inhibits IL‐1β‐induced chondrocyte inflammation and catabolism, and improves osteoarthritis by activating Nrf2.^10^ Limonin ameliorates acetaminophen‐induced hepatotoxicity by activating the antioxidant pathway of Nrf2 signaling pathway and inhibiting NF‐κB inflammation by upregulating Sirt1.^11^ In addition, Nrf2 signaling pathway is involved in INDO‐ induced gastrointestinal ulcer and injury. Benzyl isothiocyanate regulates inflammation, oxidative stress, and apoptosis through Nrf2/HO‐1 and NF‐κB signaling pathways, and acts on INDO‐induced gastric injury in rats.^12^ Autophagy deficiency reduces INDO‐induced intestinal epithelial cell injury by activating ERK/Nrf2/HO‐1 pathway.^13^

Therefore, it is hypothesized that Limonin plays a regulatory role in intestinal injury by acting on Nrf2/ARE signaling pathway.

## 2 MATERIALS AND METHODS

### 2.1 Induction of intestinal ulcer model in rats

Male Wistar rats (200–250 g) were used for experiment and were randomly divided into five groups (Control, Indomethacin (INDO), INDO + Limonin (10 mg/kg), INDO + Limonin (40 mg/kg) and Limonin (40 mg/kg) groups), containing five animals each and were under a 12/12‐h light/dark cycle for about 1 week for acclimatization before starting the experiment. Group one served as a normal control and the rats did not receive any treatment. Rats in Group two received INDO 10 mg/kg, s.c., for 9 consecutive days (negative control). Rats in Groups 3 and 4 were treated with Limonin (10 mg/kg or 40 mg/kg, p.o.,) for 4 days). Rats in Group 5 were treated with Limonin (40 mg/kg, p.o.,) for 4 days).^10^, ^14^ On 13 days, the animals were euthanized by CO_2_ asphyxiation. Blood was collected via cardiac puncture. The small intestine was gently dissected and cleaned with saline. The jejunoileal segment (10 cm from the Treitz ligament till 10 cm from the ileocecal junction) was divided into two parts. Scraping off the mucosa of one jejunoileal section was done, and the snap was frozen using liquid nitrogen and preserved at −80°C for further use. The other section was fixed with 10% formalin and embedded in paraffin for Hematoxylin and eosin (HE) staining. All animal procedures were operated in light of the NIH Guide for the care and use of laboratory animals approved by the ethical guidelines of Air Force Specialty Medical Center (approval number: 22–43), and were conducted in light of the ARRIVE guidelines.

### 2.2 Evaluation of peptic ulcer lesions

Scoring of ulcers was performed according to the criteria: 0 for normal colored stomach, 0.5 for red coloration, 1 for spot ulcer, 1.5 for hemorrhagic streaks, 2 for ulcer between 3 and 5 mm^2^, and 3 for ulcer >5 mm^2^. Mean ulcer score for each animal was expressed as ulcer index.

Ulcer index = (UN + US + UP) × 10^−1^.

Where UN is the average of number of ulcers per animal, US is the average of severity score, and UP is the percentage of animal with ulcer.

### 2.3 HE staining

The histopathological examination of the intestinal tissues was evaluated by HE staining. The intestinal tissues were fixed with 4% formalin solution, dehydrated in different concentration of alcohol, and embedded in paraffin. Then they were cut into 4 µm paraffin section and detected by HE staining.

### 2.4 Cytokine analysis by ELISA

The activity of interleukin‐6 (IL‐6), IL‐1β, and tumor necrosis factor‐α (TNF‐α) in serum of rats was detected using ELISA kits according to the manufacturer's protocols (Nanjing Jiancheng).

### 2.5 Assessment of myeloperoxidase (MPO) activity

According to manufacturer's instructions, the MPO activity in the proteins extracted from intestinal tissues was assessed.

### 2.6 Detection of oxidative stress indexes

The activity of superoxide dismutase (SOD), glutathione peroxidase (GSH‐Px), catalase (CAT), and lipid peroxidation (LPO), and the concentration of malondialdehyde (MDA) were measured in rat intestinal tissues with the corresponding kits (Nanjing Jiancheng).

### 2.7 Immunohistochemical (IHC)

Following the deparaffinization and rehydration of paraffin‐embedded intestinal tissues Section (4‐μm thick), 3% H_2_O_2_ was used to diminish endogenous peroxidase activity. Next, antibodies and biotin‐labeled goat anti‐mouse/rabbit Immunoglobulin G secondary antibodies were supplemented to the sections soaked in normal goat serum seal solution. Nuclear staining with DAB (Sigma Aldrich) was performed and hematoxylin was to counterstain the sections. IHC was performed using a kit (Beijing Zhong Shan Jin Qiao Biotechnology) according to the instructions.

### 2.8 Western blot

The RIPA buffer (Sigma‐Aldrich) was used to extract total protein in the cells and the intestinal tissues. The BCA protein quantitative assay kit (Biosharp) was used to determine the protein concentration. Thirty micro gram proteins were loaded into 10% sodium dodecyl sulfate gels for separation and transferred to polyvinylidene fluoride membranes. Then 5% nonfat milk powder was used to block the membranes, which were incubated overnight at 4°C with primary antibodies. Subsequently, the membranes were incubated with horseradish peroxidase‐conjugated secondary antibodies for 2 h at 37°C. The expression levels of the different proteins were detected using enhanced chemiluminescence reagent (Bio‐Rad Laboratories, Inc.). Proteins bands were visualized using enhanced chemiluminescence (Thermo Fisher Scientific, Inc.). The data were analyzed using ImageJ 1.52 k software (version 1.46; National Institutes of Health).

### 2.9 Molecular docking

The structure of Limonin was drawn in the ChemDraw software, and then imported into OpenBabel software (v2.2.1) for hydrogenation and converted into a mol2 format file. The structure of Nrf2 was downloaded from the RCSB Protein Data Bank webpage (https://www.rcsb.org/). The protein Protein Data Bank file was opened in PyMOL software (v2.2.0) to remove the excess water molecules, delete any irrelevant small ligands originally carried, and only keep the protein structure. Since the downloaded protein structure comes with a ligand, the original ligand was deleted and the original ligand position was set as the docking site. After the running in AutoDock (v4.2) was completed, the specific docking energy values were displayed. And protein–ligand interaction profiler (PLIP; https://plip-tool.biotec.tu-dresden.de/plip-web) was used to analyze the results.

### 2.10 Cell culture

Rat small intestinal epithelial cell line IEC‐6 cells were maintained in Dulbecco's modified Eagle medium supplemented with 10% fetal bovine serum (FBS; Gibco) at 37°C in 5% CO_2_. After the different concentrations of Limonin (MedChemExpress, HY‐17411, 25, 50, and 100 μM) were added for 24 h of incubation, different concentrations of INOD (MedChemExpress, HY‐14397, 300, 320, and 350 μM) were supplemented for 24 h. In addition, before INDO induction for 24 h, cells were pretreated with different concentrations of Limonin for 6 h. Ten micrometer Nrf2 inhibitor ML385 (MedChemExpress; HY‐100523) was added for pretreatment for 24 h.

### 2.11 Cell viability assay

After the cells were given different treatments, each well was treated with 10 μL CCK‐8 and incubated for 3 h at 37°C according to the manufacturer's instructions. Subsequently, the density of the O.D. was recorded by a spectrophotometer (Thermo Fisher) at 450 nm.

### 2.12 TUNEL staining assay

IEC‐6 cell apoptosis was analyzed using In Situ Cell Death Detection Kit (Roche Diagnostics GmbH). Briefly, after being fixed with 4% paraformaldehyde, IEC‐6 cells were incubated with 0.1% Triton X‐100 for 0.5 h for permeabilization and were then treated in TUNEL reaction mixture for 1 h at 37°C. Morphological assessment was performed by fluorescence microscopy.

### 2.13 Measurement of reactive oxygen species (ROS)

ROS generation was detected by way of 2′,7′‐dichlorodihydrofluorescein diacetate (DCFH‐DA) (mlbio) kit. Briefly, after treated, IEC‐6 cells were maintained with 10 μM DCFH‐DA for 0.5 h in the dark at 37°C. Then, the images were photographed by means of a fluorescence microscope (80i, Nikon).

### 2.14 Immunofluorescence confocal microscopy

To investigate the expression of ZO‐1 protein, the treated IEC‐6 cells on coverslips were fixed, permeabilized, blocked, and then incubated with ZO‐1 primary antibody overnight and secondary antibodies. After being stained with 4′,6‐diamidino‐2‐phenylindole for 20 min, cells were photographed with an Olympus confocal microscope.

### 2.15 Statistical analysis

All data were expressed as mean ± SD. One‐way analysis of variance (ANOVA) with Tukey's post hoc test was performed for multiple comparisons with GraphPad Prism 5 software. The *p* < .05 was viewed to be statistically significant. Each experiment was conducted at least three times.

## 3 RESULTS

### 3.1 Limonin improves intestinal injury and ulcer induced by INDO in rats

**Figure 1:**
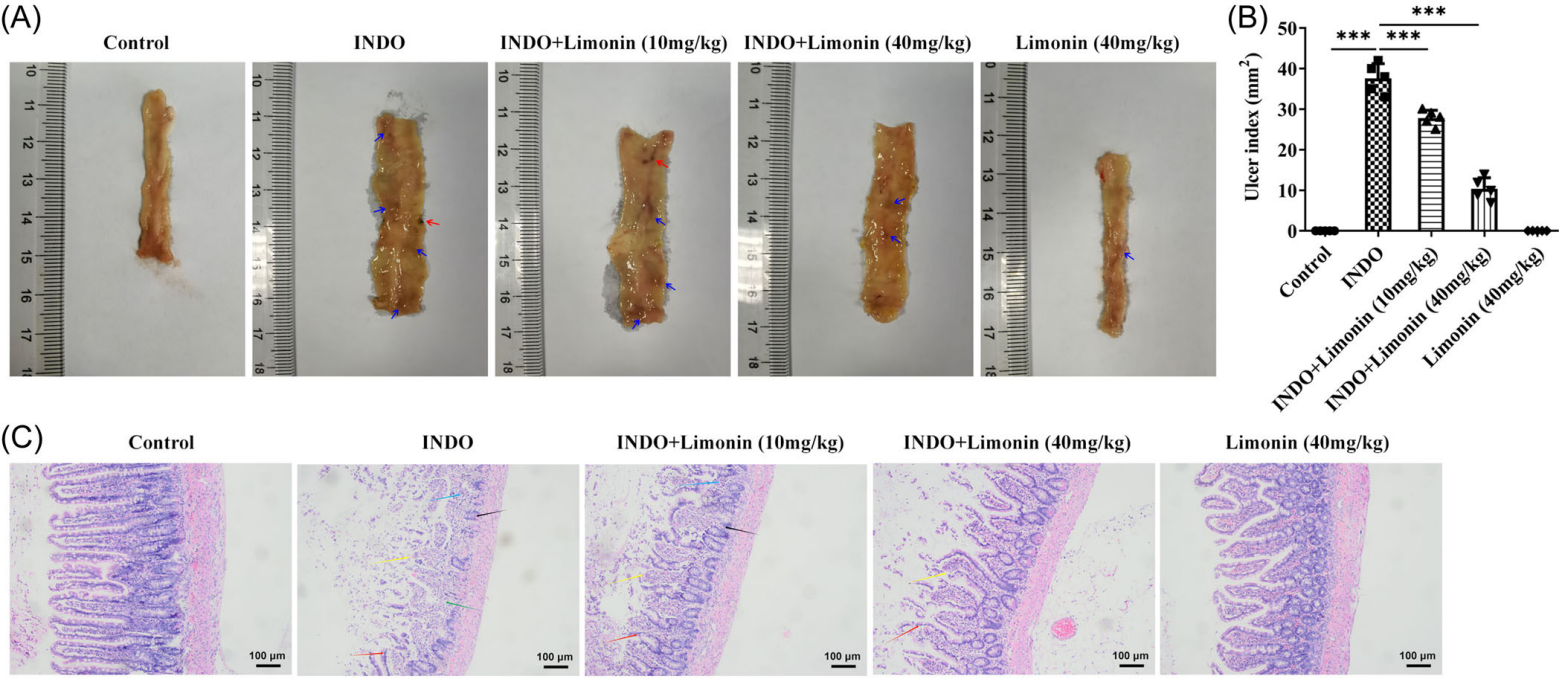
Limonin improves intestinal injury and ulcer induced by INDO in rats. (A) Photograph of the small intestine of rats. (B) The ulcer index of rats. (C) Hematoxylin and eosin staining was used to detect the pathological damage of small intestinal tissues. Magnification 100, *N* = 5, analysis of variance with Tukey's post hoc test, ***p < .001. INDO, indomethacin.

The damage of the intestine mucosa and the ulcer index of the small intestine were evaluated. The results showed that the damage of the intestine mucosa was serious and the ulcer index was increased after the induction of INDO. However, after the administration of Limonin, the intestinal mucosal injury was alleviated and the ulcer index was decreased. In addition, rats treated with 40 mg/kg Limonin alone showed no significant damage to the intestinal mucosa (Figure 1A,B). HE staining was used to detect the pathological damage of small intestinal tissues, and the results were consistent with the trend of ulcer index (Figure 1C).

### 3.2 Limonin improves inflammatory response and oxidative stress induced by INDO in rats

**Figure 2:**
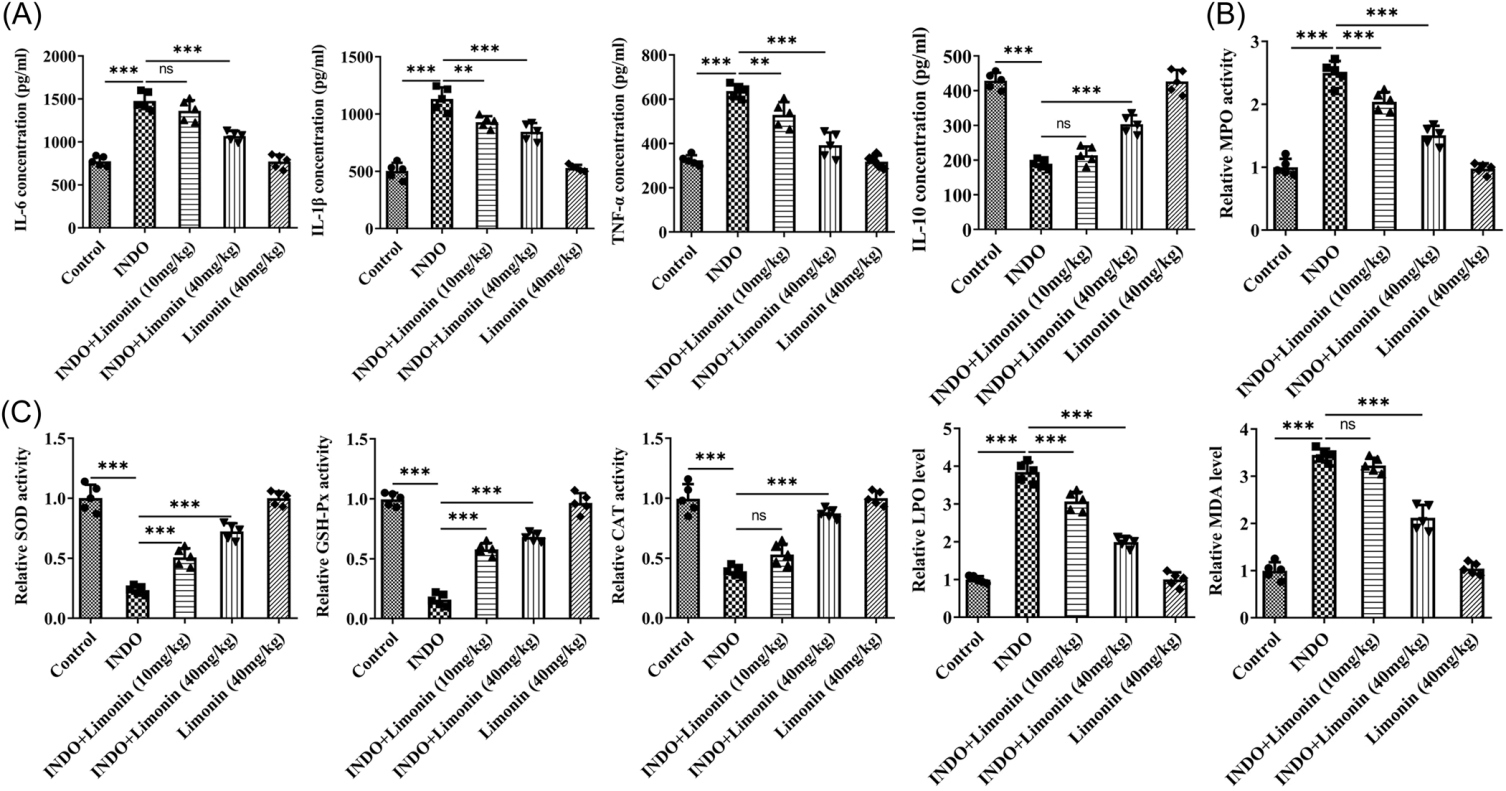
Limonin improves inflammatory response and oxidative stress induced by INDO in rats. (A) The levels of serum inflammatory factors were detected by ELISA. (B) The kit was used to detect the activity of MPO in small intestinal tissues. (C) The kits were used to detect the levels of oxidative stress‐related factors in small intestinal tissues. *N* = 5, analysis of variance with Tukey's post hoc test, **p < .01, ***p < .001. CAT, catalase; GSH‐Px, glutathione peroxidase; INDO, indomethacin; LPO, lipid peroxidation; MPO, myeloperoxidase; SOD, superoxide dismutase; TNF, tumor necrosis factor.

The levels of serum inflammatory factors were detected by ELISA. The results showed that the activity of IL‐6, IL‐1β, and TNF‐α in INDO group was significantly increased compared with those in control group, while the activity of IL‐10 was increased. Compared with INDO group, the activity of IL‐6, IL‐1β, and TNF‐αin INDO + Limonin (10 mg/kg) and INDO + Limonin (40 mg/kg) groups was significantly decreased, while the activity of IL‐10 was decreased. There was no significant change in the activity of inflammatory factors in Limonin (40 mg/kg) group compared with the control group (Figure 2A). Subsequently, the kit was used to detect the activity of MPO in small intestinal tissues, and it was found that the activity of MPO was increased significantly after induction of INDO and decreased significantly after further administration of Limonin (Figure 2B). Compared with the control group, the activity of SOD, GSH‐Px and CAT in the INDO group was significantly decreased, while the activity of LPO and MDA was significantly increased. Compared with INDO group, the activity of SOD, GSH‐Px, CAT, LPO, and MDA was reversed after Limonin administration (Figure 2C).

### 3.3 Limonin improves intestinal barrier induced by INDO in rats

**Figure 3:**
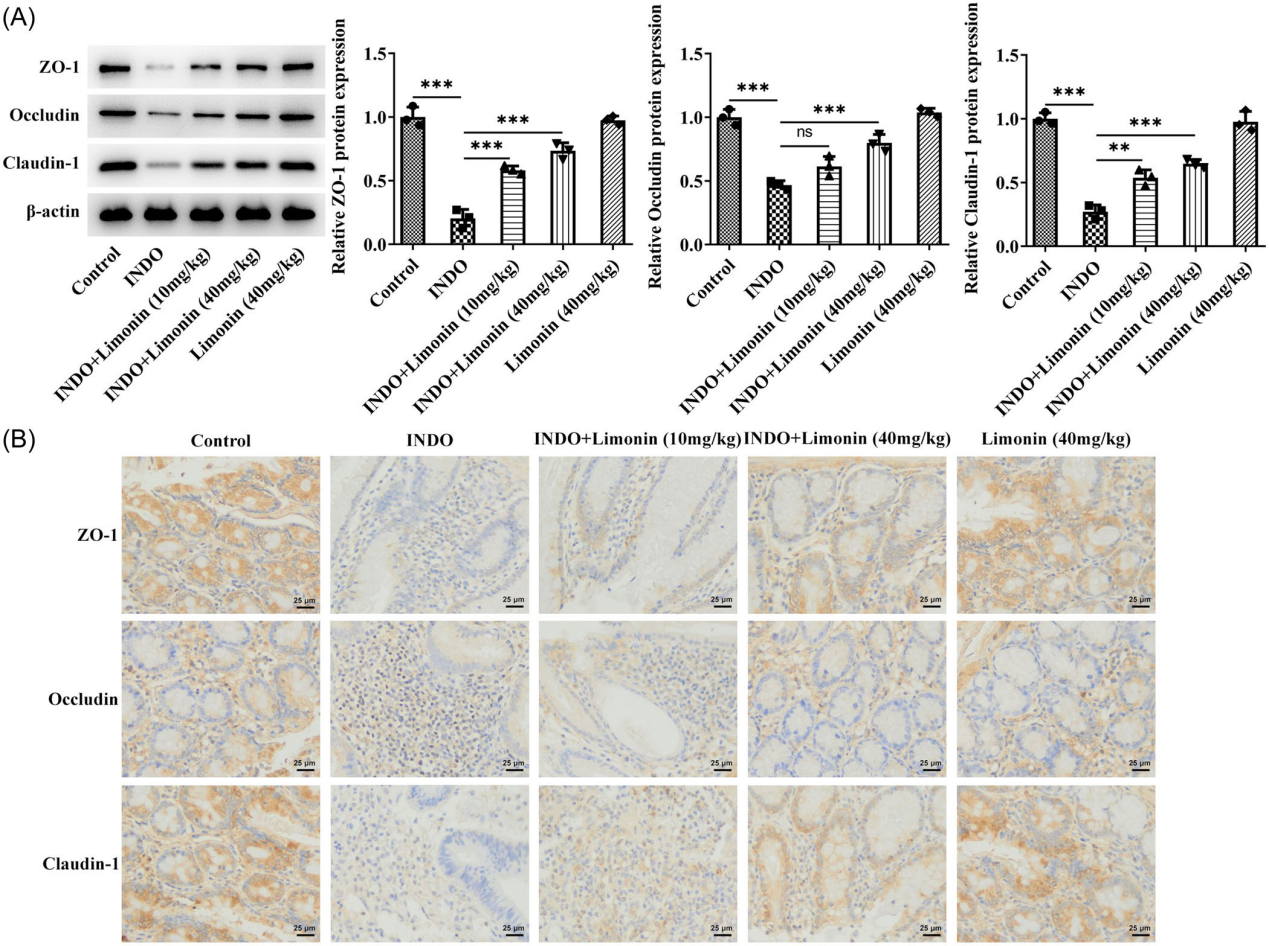
Limonin improves intestinal barrier induced by INDO in rats. Western blot (A) and immunohistochemical (B) were used to detect the expressions of tight junction proteins ZO‐1, Occludin and claudin‐1 in small intestinal tissues. Magnification 400, *N* = 3, analysis of variance with Tukey's post hoc test, **p < .01, ***p < .001. INDO, indomethacin.

Western blot and IHC were used to detect the expression of tight junction proteins ZO‐1, Occludin, and claudin‐1 in small intestinal tissues. The results showed that the expression of ZO‐1, Occludin, and claudin‐1 in INDO group was significantly decreased compared with the control group. After further administration of Limonin, the expression of ZO‐1, Occludin, and claudin‐1 was significantly increased (Figure 3A,B).

### 3.4 Limonin regulates the Nrf2/ARE pathway

**Figure 4:**
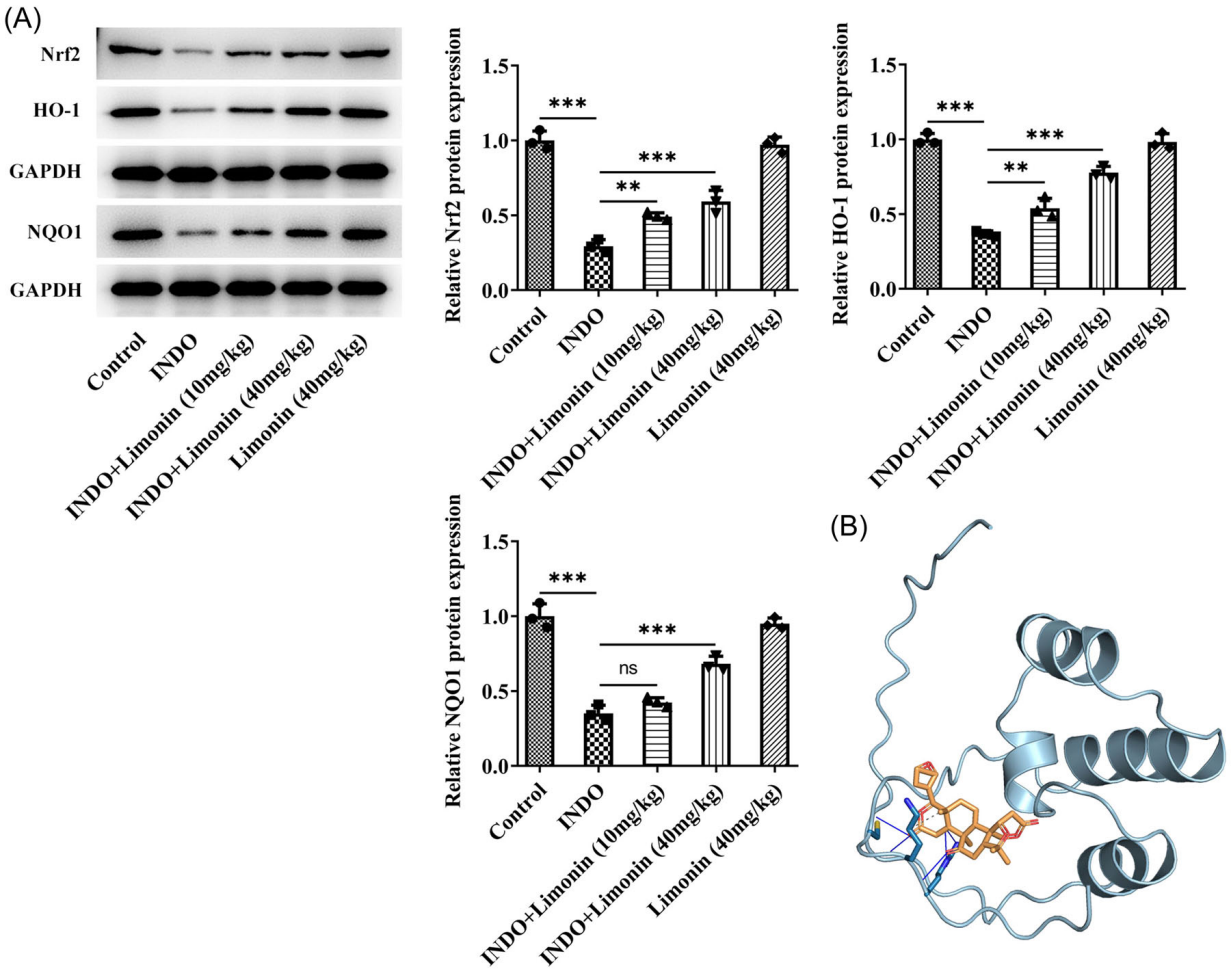
Limonin regulates the Nrf2/ARE pathway. (A) The expression of Nrf2/ARE pathway‐related proteins Nrf2, HO‐1, and NQO1 was detected by Western blot. (B) Molecular docking plot of Limonin with Nrf2. *N* = 3, analysis of variance with Tukey's post hoc test, **p < .01, ***p < .001. INDO, indomethacin.

The expression of Nrf2/ARE pathway‐related proteins Nrf2, HO‐1, and NQO1 was detected by Western blot. The results showed that compared with the control group, the expression of Nrf2, HO‐1, and NQO1 in the INDO group was significantly decreased. Nrf2, HO‐1, and NQO1 expression were reversed in INDO + Limonin (10 mg/kg) and INDO + Limonin (40 mg/kg) groups compared with INDO group (Figure 4A). In addition, it was also found that Limonin could dock with Nrf2 (Figure 4B).

### 3.5 The Nrf2 inhibitor ML385 reverses the protective effect of Limonin on INDO‐induced IEC‐6 cells in vitro

**Figure 5:**
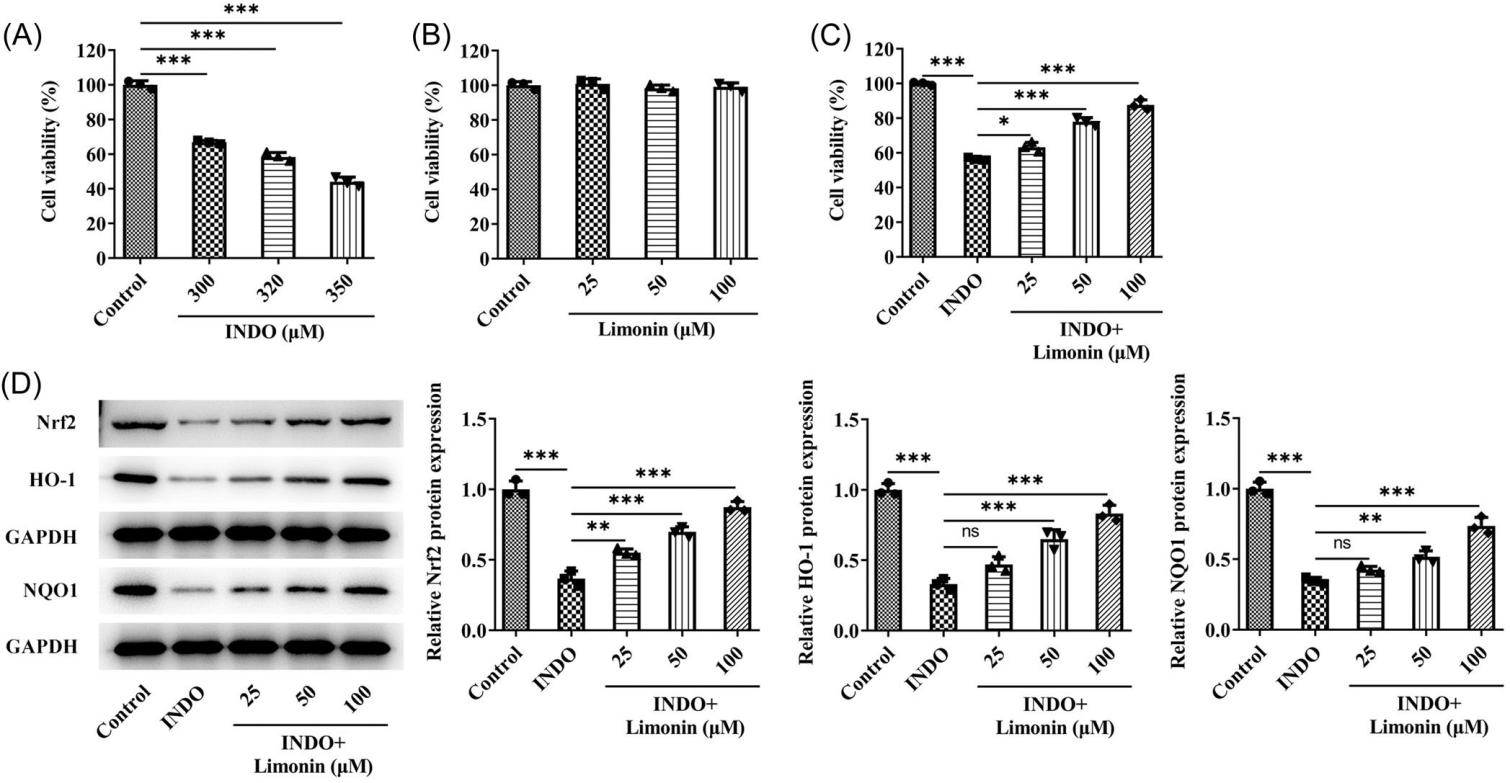
Limonin regulates the Nrf2/ARE pathway. (A) CCK8 was used to detect cell viability after induction of different concentrations of INDO. (B) CCK8 was used to detect cell activity after treatment with different concentrations of Limonin. (C) CCK8 was used to detect cell activity after treatment with different concentrations of INDO and Limonin. (D) Western blot was used to detect the expressions of tight junction proteins ZO‐1, Occludin, and claudin‐1 in cells. *N* = 3, analysis of variance with Tukey's post hoc test, *p < .05, **p < .01, ***p < .001. INDO, indomethacin.

**Figure 6:**
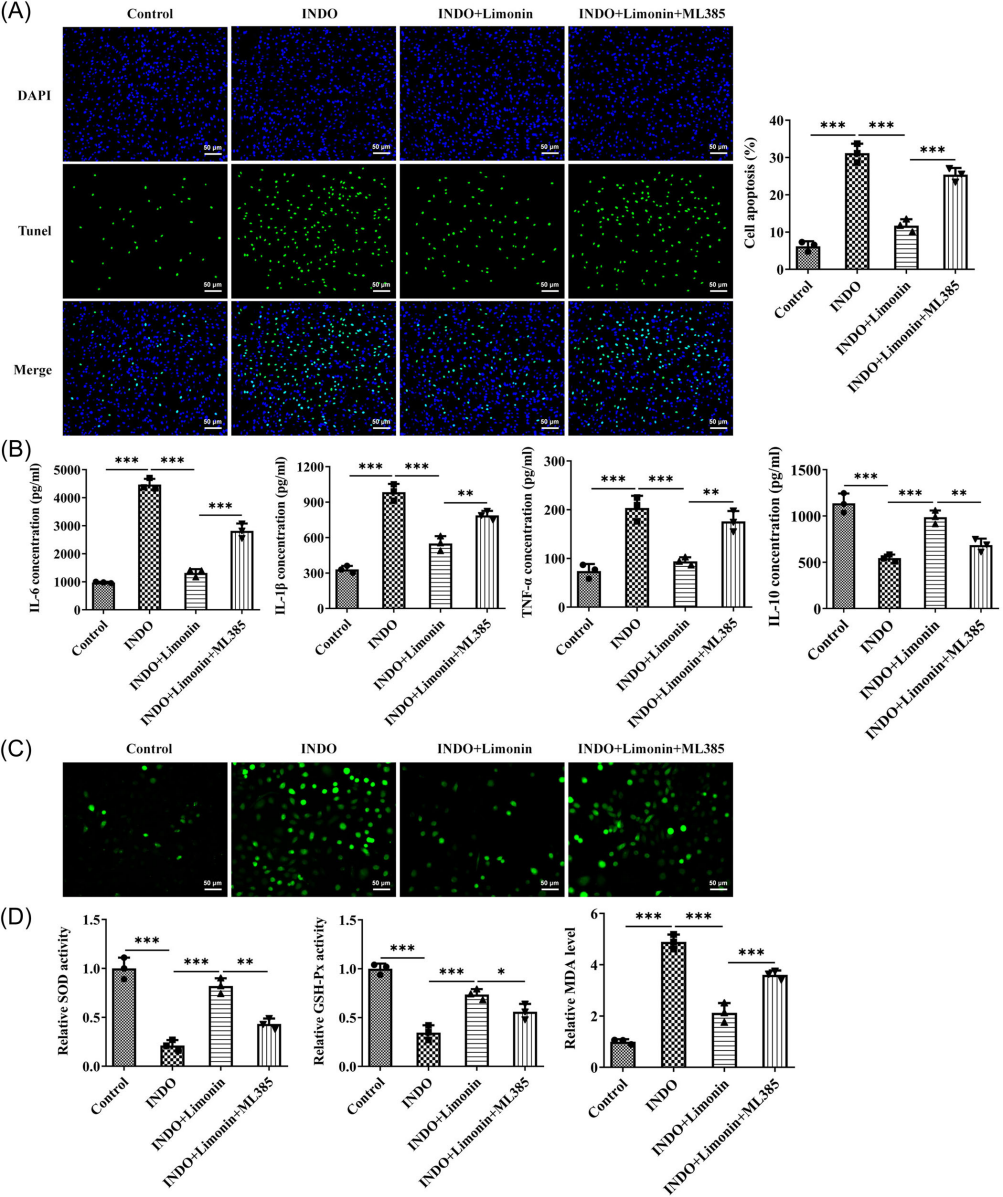
The Nrf2 inhibitor ML385 reverses the inhibition of inflammatory response and oxidative stress of Limonin on INDO‐induced IEC‐6 cells in vitro. (A) Apoptosis was detected by TUNEL assay. Magnification 200. (B) The levels of serum inflammatory factors were detected by ELISA. (C) 2′,7′‐dichlorodihydrofluorescein diacetate fluorescent probe detected the reactive oxygen species level in cells. Magnification 200. (D) The kits were used to detect the levels of oxidative stress‐related factors in cells. *N* = 3, analysis of variance with Tukey's post hoc test, *p < .05, **p < .01, ***p < .001. INDO, indomethacin.

**Figure 7:**
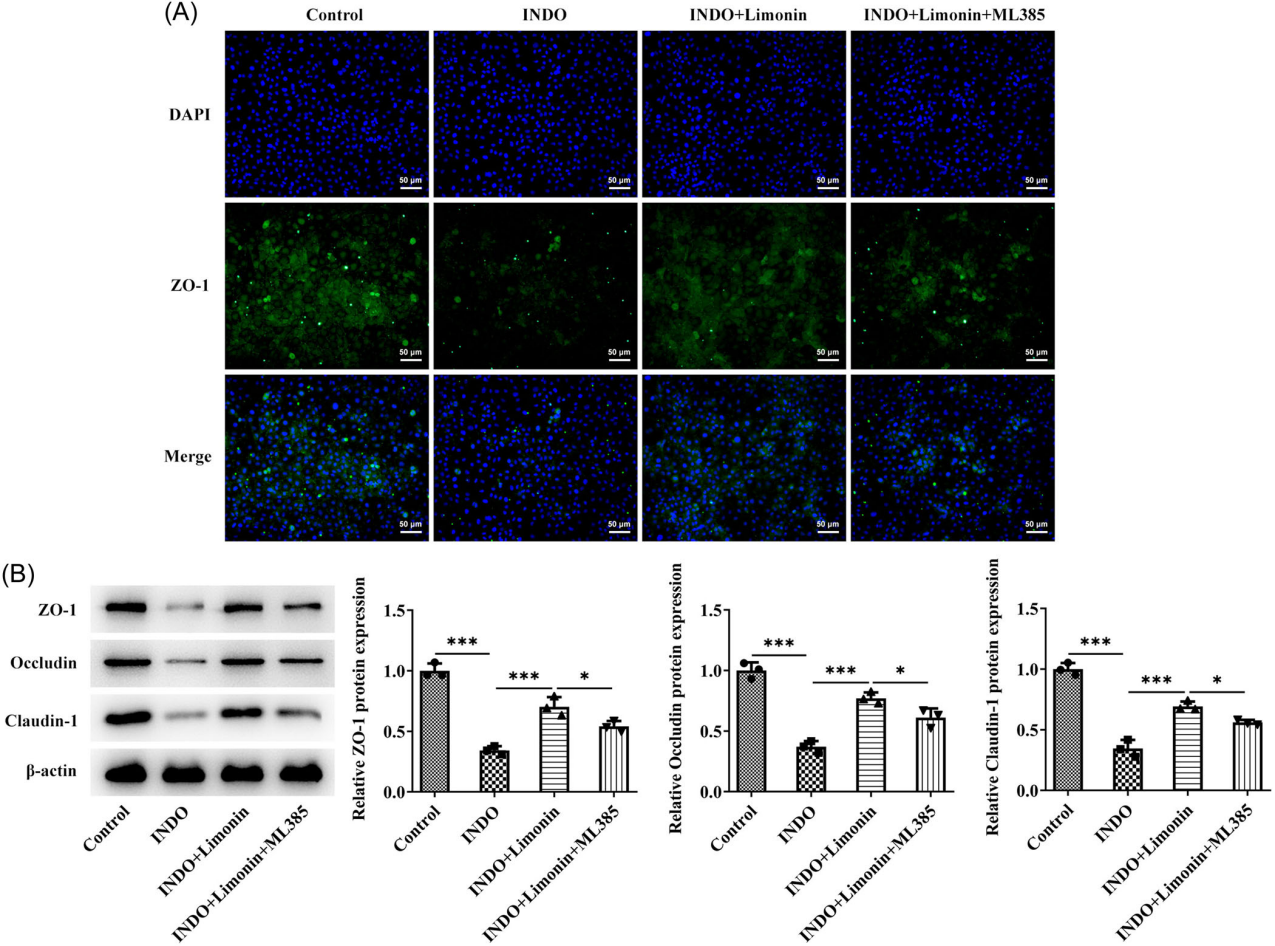
The Nrf2 inhibitor ML385 reversed the protective effect of Limonin on INDO‐induced IEC‐6 cells in vitro. (A) The expression of ZO‐1 was detected by IF assay. Magnification 200. (B) Western blot was used to detect the expressions of tight junction proteins ZO‐1, Occludin and claudin‐1 in cells. *N* = 3, analysis of variance with Tukey's post hoc test, *p < .05, **p < .01, ***p < .001. DAPI, 4′,6‐diamidino‐2‐phenylindole; INDO, indomethacin.

IEC‐6 cells were induced by INDO to further explore the regulatory mechanism of Limonin on intestinal ulcer. CCK8 was used to detect cell viability after induction of different concentrations of INDO. The results showed that compared with the control group, the cell activity was decreased in a dose‐dependent manner (Figure 5A). Then 320 μM INDO was selected for subsequent modeling. Subsequently, CCK8 was used to detect cell activity after treatment with different concentrations of Limonin, and the results showed that Limonin did not damage cells (Figure 5B). Then, the cells were grouped into control, INDO, INDO + 25 μM Limonin, INDO + 50 μM Limonin, and INDO + 100 μM Limonin groups. CCK8 results showed that Limonin reversed the inhibitory effect of INDO on cell activity in a concentration‐dependent manner (Figure 5C). Western blot results showed that Limonin reversed the inhibitory effect of INDO on Nrf2/ARE pathway‐related proteins in cells in a concentration‐dependent manner (Figure 5D). Hundred‐micrometer Limonin was selected for subsequent experiments, and Nrf2 inhibitor ML385 was adopted. The cells were divided into control, INDO, INDO + Limonin, and INDO + Limonin + ML385 groups. Apoptosis was detected by TUNEL, and the results showed that apoptosis was significantly increased after INDO induction, while Limonin could reverse the apoptosis induced by INDO. In addition, compared with the INDO + Limonin group, the apoptosis of cells in the INDO + Limonin + ML385 group was significantly increased (Figure 6A). ELISA results showed that the activity of IL‐6, IL‐1β, and TNF‐α in the INDO + Limonin + ML385 group was significantly increased compared with those in the INDO + Limonin group, while the activity of IL‐10 was decreased (Figure 6B). DCFH‐DA fluorescent probe detected the ROS level in cells, and it was found that INDO could induce the increase of ROS in cells. However, Limonin could reverse the increase of ROS induced by INDO. Compared with the INDO + Limonin group, the activity of ROS in the INDO + Limonin + ML385 group was increased (Figure 6C). Subsequently, the levels of oxidative stress‐related factors were detected, and the results showed that the trend of MDA was consistent with that of ROS, while the trend of SOD and GSH‐Px was opposite to that of ROS (Figure 6D). IF results showed that compared with INDO + Limonin group, the expression of ZO‐1 in INDO + Limonin + ML385 group was significantly decreased (Figure 7A). Western blot results showed that the expression of ZO‐1, Occludin, and claudin‐1 was consistent with the trend of IF results of ZO‐1 (Figure 7B).

## 4 DISCUSSION

Intestinal injury and ulcer is a common clinical lesion, to which NSAIDs are the common contributing factors. It has been reported that erosive or ulcerative lesions of the intestinal mucosa are found in 76.3% of capsule endoscopy subjects taking NSAIDs.^15^ Therefore, in this paper, INDO was used to induce intestinal mucosal injury of rats. Limonin is a triterpene aglycone derivative with a variety of pharmacological properties, including anticancer, anti‐inflammatory, antibacterial, and antiviral activities.^16^ Previous study has shown that Limonin inhibits the development of intestinal polyps in adenomatous polyposis coli (Apc)‐mutant mice and may improve intestinal carcinogenesis.^17^ However, the intervention means of small intestinal lesions are less studied. In this study, the effect and mechanism of Limonin on intestinal mucosal injury induced by INDO in rats were investigated. Our study found that Limonin could significantly inhibit INDO‐induced intestinal mucosal injury and reduce the occurrence of intestinal ulcers in rats. Moreover, Limonin could significantly reduce INDO‐induced inflammation and oxidative stress in rats. Study has shown that Limonin can modulate immune and inflammatory responses to inhibit mouse colorectal adenocarcinoma model.^18^ In addition, Limonin derivatives regulate inflammation by inhibiting TLR4/NF‐κB signaling pathway.^19^ Moreover, Limonin has antioxidant and anti‐inflammatory effects on rat ischemic liver, thereby protecting hepatocytes from I/R injury.^20^ Limonin alleviates nonalcoholic fatty liver disease by reducing lipid accumulation, suppressing inflammation and oxidative stress.^21^ The above results showed that Limonin could significantly inhibit the inflammatory response and oxidative stress response in the disease, which was consistent with our experimental results. Tight junctions and adhesion junctions, as the main cell junctions, are the key components of intestinal mucosal epithelial barrier and play an important role in maintaining intestinal barrier function.^22^ In patients with functional dyspepsia, tight junction proteins in duodenal mucosa are significantly reduced, resulting in intestinal mucosal barrier damage and thus intestinal mucosal injury.^23^ In our experiment, we found that the expression of tight junction‐related proteins ZO‐1, Occludin, and Claudin‐1 decreased significantly after INDO induction. The study of Jung et al. proved that the expression of Occludin and ZO‐1 proteins in IEC‐6 cells induced by INDO decreased significantly, which was also consistent with our results.^24^ Limonin could significantly activate the expression of tight junction proteins ZO‐1, Occludin, and claudin‐1 induced by INDO, thereby improving intestinal barrier function in rats.

**Figure 8:**
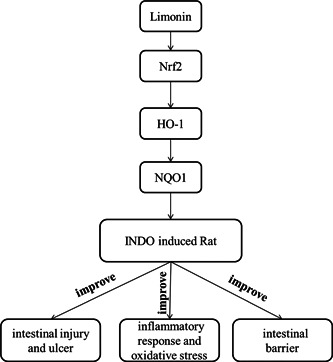
Mechanism diagram. INDO, indomethacin.

Next, we discussed the regulatory mechanism of Limonin. The study has shown that Limonin has a protective effect on doxorubicin‐induced cardiotoxicity through Nrf2 signaling pathway.^14^ Limonin significantly upregulates hepatic Nrf2/HO‐1 signaling and reverses the reduction of glutathione and accumulation of ROS in nonalcoholic fatty liver disease.^21^ In our paper, we found that Limonin could dock with Nrf2 molecules and activate Nrf2/ARE signaling pathway in INDO‐induced intestinal epithelial IEC‐6 cells. It has been shown to protect the small intestine from NSAID‐induced damage by upregulating the Nrf2‐keap1‐dependent antioxidant system and inhibiting the invasion of the mucosa by anaerobic bacteria.^25^ Moreover, by regulating Nrf2 pathway and NF‐κBp65 pathway, INDO‐induced enteritis in rats and IEC‐6 cell damage were significantly prevented.^26^ These results indicate that Nrf2/ARE signaling pathway plays an important role in INDO‐induced intestinal injury, and Limonin can play a role in disease improvement by regulating Nrf2/ARE signaling pathway. Further addition of Nrf2 inhibitor ML385 could significantly reverse the protective effect of Limonin on IEC‐6 cells induced by INDO in vitro. These results indicated that Limonin could ameliorate intestinal mucosal injury induced by INDO via activating Nrf2/ARE signaling pathway (Figure 8).

## 5 CONCLUSION

In conclusion, we found that Limonin ameliorated INDO‐induced intestinal damage and ulcers through Nrf2/ARE pathway. Our paper provides a theoretical basis for clinical treatment of intestinal injury and ulcer caused by drug action with Limonin.

## AUTHORS CONTRIBUTIONS

Zhilei Tian and Bo Jia contributed to the conception and design of the present study, analyzed and interpreted the data, and critically revised the manuscript for important intellectual content. Leyi Zhao, Pengpeng Liu, and Meng Li contributed to designing the study and analyzed the data. Zhilei Tian and Bo Jia drafted and revised the manuscript. Zhilei Tian confirmed the authenticity of all the raw data. The final manuscript has been read and approved by all authors.

## CONFLICT OF INTEREST STATEMENT

The authors declare no conflict of interest.

## Data Availability

The datasets used and/or analyzed generated during the current study are available from the corresponding author on reasonable request.
